# Supplementary material for: Clinical Profiles and Factors Associated with a Low Sodium Intake in the Population: An Analysis of the Swiss Survey on Salt
Source: Nutrients. 2020 Nov 23;12(11):3591. doi: 10.3390/nu12113591 (PMC7700385; doi:10.3390/nu12113591)

Clinical profiles and factors associated with a low sodium intake in the population: an analysis of the Swiss Survey on Salt

Michel Burnier, Fred M. Paccaud, Murielle Bochud

Supplemental material

Supplemental table 1: Beliefs about the effects of salt on health

	5 g or more n=1329	Less than 5g n=218	P value (chi2)
Impact on health	%	%	0.50
Don't know	12.88	10.14	
No	10.82	11.98	
Yes	76.30	77.88	
Impact on:			
Hypertension, % yes	79.38	80.28	0.76
Heart disease, % yes	40.56	41.74	0.74
Myocardial infarction, % yes	21.60	16.51	0.09
Stroke, % yes	21.82	17.89	0.19
Obesity, % yes	23.70	26.15	0.43
Diabetes, % yes	7.60	5.50	0.27
Irritable bowel disease, % yes	7.07	4.59	0.17
Tuberculosis, % yes	0.53	0.46	0.90
Other diseases, % yes	9.03	9.17	0.95
No disease, % yes	4.97	3.21	0.26

Supplemental Table 2: Socio-demographic and lifestyle characteristics of Swiss women eating more or less than 5 g of salt per day

	≥5 g (n=594)	< 5 g (n=151)	P (χ^2)
Civil status (%)			0.37
Single	39.1	37.1	
Married	38.6	35.1	
Other*	22.4	27.8	
Nationality (% Swiss)	89.1	90.7	0.55
Born in Switzerland (% yes)	78.3	84.1	0.11
Linguistic region (%)			0.04
French	29.1	39.7	
German	56.4	48.3	
Italian	14.5	11.9	
Education level (%)			0.72
Low (mandatory or less)	16.5	15.2	
Medium (apprenticeship/high school)	45.0	51.7	
High (University, specialist training, high specialist training)	35.5	33.1	
Living alone in household (% yes)	72.7	68.2	0.27
Current smoker (% yes)	16.3	14.6	0.60
Current alcohol use (% yes)	78.8	68.9	0.01
Alcohol consumption, frequency (%)			0.36
.. Never	19.8	27.0	
.. Less than once per week	34.8	30.4	
1-2 times per week	29.9	27.0	
< 1x/day, more than 2x/week	7.3	6.0	
at least once per day	8.2	9.5	
Physical activity level			0.93
Nearly never	18.0	19.9	
Less than once per week	14.8	13.3	
Once per week	18.2	17.9	
More than once per week	49.0	49.0	

*Other means divorced, separated or widowed.

Supplementary table 3. Participants' anthropometric and biological characteristics by salt intake category (more or less than 5 g NaCl per 24h) among women only

		≥ 5g		< 5 g	
Variable	N	Mean (SD)	N	Mean (SD)	P value
Urine Na excretion (g/24h)	594	8.88 (3.0)	151	3.81 (1.0)	<0.001
Age (years)	594	45.5 (17.5)	151	48.8 (20.7)	0.04
BMI (kg/m^2)	594	24.7 (4.8)	151	22.6 (3.7)	<0.001
Body weight (Kg)	594	66.7 (15.7)	151	61.0 (9.7)	<0.001
Body height (cm)	594	164.6(6.6)	151	164.3 (6.8)	0.62
Menopause (%)	578	39.3	148	47.3	0.08
Contraceptive pill (%) (premenopausal women only)	342	31.0	76	34.2	0.59
Systolic BP (mm Hg)	593	118 (15)	150	118 (16)	0.61
Diastolic BP (mm Hg)	593	72.1 (9.6)	150	70.9 (8.3)	0.13
Heart rate (b/min)	594	72.8 (10.9)	151	75.1 (11.9)	0.02
Serum creatinine	542	70.5 (11.1)	135	72.9 (13.3)	0.04
Serum K	543	4.15 (0.41)	135	4.12 (0.37)	0.41
Serum Na	543	141.6 (2.0)	135	141.6 (2.3)	0.90
Serum Ca	543	2.29 (0.10)	135	2.28 (0.10)	0.67
Serum protein	541	71.0 (4.1)	135	71.0 (5.1)	0.95
Serum urea	542	5.07 (1.40)	135	4.97 (1.74)	0.51
eGFR using CKD epi	542	91.1 (18.7)	135	87.7 (22.7)	0.03
Serum uric acid	543	257 (61)	135	260 71)	0.64
Urine urea excretion (mmol/24h)	594	326 (98)	151	221 (73)	<0.001
Estimated protein intake (g/24h)	594	70.0 (11.1)	151	50.5 (13.2)	<0.001
Urine K excretion (mmol/24h)	594	62.3 (20.9)	151	45.7 (20.6)	<0.001
Urine creatinine excretion (mmol/kg/24h)	594	0.16 (0.04)	151	0.13 (0.04)	<0.001
Urine volume (mL/24h)	594	2110 (916)	151	1547 (815)	<0.001

Supplementary table 4. Behavioral characteristics in women eating more or less than 5 g NaCl per day (questionnaire data).

	N	≥ 5 g n=594 (%)	<5g, n=151 (%)	P value (chi2)
Consumption of fruits	737			0.61
Less than once per day		18.7	20.0	
1-2 portions per day		52.5	48.0	
3 or more portions per day		28.8	32.0	
Consumption of vegetables	740			0.28
Less than once per day		14.6	19.9	
1-2 portions per day		57.9	55.0	
3 or more portions per day		27.5	25.2	
Doing most of cooking at home	745	81.5	82.8	0.71
Caffeine, % 4 or more cups/d	744	25.0	16.0	0.02
Paying attention to diet	744	74.9	70.9	0.32
Diet in the past 12 months	744	14.8	15.9	0.75
Usual quantity of non-alcohol drinks per day, mean(SD) #	710	1.72 (0.61)	1.64 (0.65)	0.11#
Self-estimated salt consumption				<0.001
Low		31.5	46.4	
Medium		58.0	51.7	
High		10.6	2.0	
Number of days/week consuming meat (%)	745			0.03
0-1		19.2	29.8	
2-3		40.2	37.8	
4-5		32.0	24.5	
6 or more		8.6	8.0	
Number of days/week consuming fish (%)	1443			0.09
Less than 1		42.4	51.0	
1		34.9	33.1	
2 or more		22.7	15.9	
Number of minutes walked per day, median (IQR)	1426	30 (20-60)	30 (20-60)	0.48*

* test for median, # T test

Supplemental figure 1:

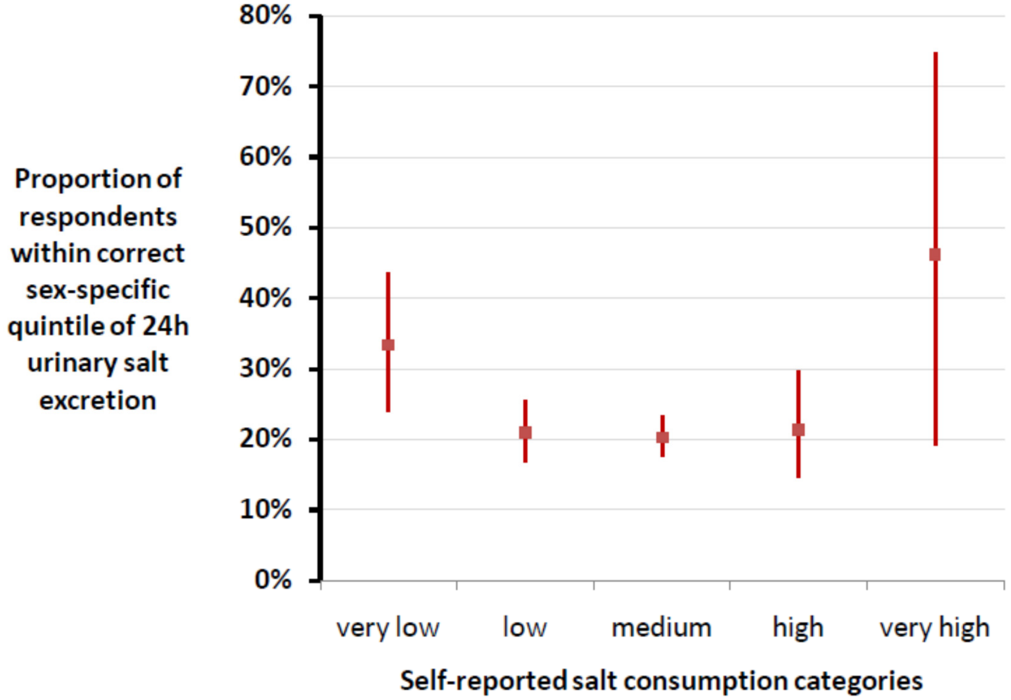

Supplement: Supplementary file 1 [file nutrients-12-03591-s001.pdf]
